# Sleep, Dreaming, and Insanity
*Sleep and Dreams. Two Lectures delivered at the Bristol Literary and Philosophical Institution. By John Addington Symonds, M.D., Consulting Physician to the Bristol General Hospital. 8vo. 1851.Sleep Psychologically considered, with Reference to Sensation and Memory. By Blanchard Fosgate, M.D., Physician to the New York State Prison at Auburn. 8vo, pp. 188. 1850.What is Mesmerism? An Attempt to explain its Phenomena, &c. By A. Wood, M.D., &c. Edinburgh: 1851.The Mesmeric Mania of 1851, &c. By J. Hughes Bennett, M.D., &c. &c. Edinburgh: 1851.


**Published:** 1851-10-01

**Authors:** 


THE JOURNAL

OF

### PSYCHOLOGICAL MEDICINE

AND

MENTAL PATHOLOGY.

OCTOBER 1, 1851.

### ART. I.—SLEEP, DREAMING, AND INSANITY.*

IF a man were always to enact his dreams he would not consider sleep to be

"Tired nature's sweet restorer."

It is the perfect repose of all cerebral action that constitutes sleep, in the strictly philosophical acceptation of the term; hence even the "stuff our dreams are made of" has a material influence on the cerebrum, and prevents a total cessation of its functions. It is true that perfect sleep is rare in this sense; and it is therefore characterized, when it occurs, as *deep* sleep, *profound* slumber, and by other phrases significative of that total cessation of cerebral activity which is its great characteristic and object.

We have said that sleep is the perfect repose of the encephalic mechanism. The merely vital mechanism goes on as usual: in some of its parts more slowly, but perhaps in others more vigorously—the repose of one system helping the activity of another. It is true that it has been called the image of death by writers of various classes, but, physiologically, it is a state of life as vigorous in its immediate sphere as the

* SLEEP AND DREAMS. Two Lectures delivered at the Bristol Literary and Philosophical Institution. By John Addington Symonds, M.D., Consulting Physician to the Bristol General Hospital. 8vo. 1851.

SLEEP PSYCHOLOGICALLY CONSIDERED, with Reference to Sensation and Memory. By Blanchard Fosgate, M.D., Physician to the New York State Prison at Auburn. 8vo, pp. 188. 1850.

WHAT IS MESMERISM? An Attempt to explain its Phenomena, &c. By A. Wood, M.D., &c. Edinburgh: 1851.

THE MESMERIC MANIA OF 1851, &c. By J. Hughes Bennett, M.D., &c. &c. Edinburgh: 1851.

waking state. It seems very probable that, during sleep, the reparation and nutrition of the organism go on much more actively than during the waking state, consequently organic or vegetative vitality is more energetic. To facilitate these objects, the active machinery of the body is stopped, or moves more slowly all that mechanism which is simply the agent of the spiritual element of our nature standing still absolutely; while the great wheels of life—those of respiration and circulation—move proportionately more slowly, and only to that extent which the activity of the organic functions requires.

It seems to be a universal law of animated nature, that repose shall follow activity. Even in vegetable life we find sleep is the rule, and in animal life we know of no exceptions. So universal a law must needs be considered to be a necessary law; hence we conclude that periodic repose or quiescence is necessary to the healthy action of organisms. There are apparent exceptions observed when we come to examine the details of special organs. Thus, for example, the heart never ceases to beat unless under abnormal circumstances. In this and other cases there is probably some compensation in the rhythmical action of the machinery; but, anyhow, during sleep the heart beats much more slowly, and there are instances on record in which its nerves became torpid with the cerebro-spinal system, so that the sufferer began to sleep the sleep of death so soon as his eyelids drooped, requiring a constant watcher at his bedside to awake him from his perilous slumber.

We do not think it necessary to detail the ordinary phenomena of sleep *seriatim*, but rather prefer to occupy our pages with an investigation of the nature of the changes which occur in the cerebral system during sleep, with reference to the phenomena of dreaming in particular, and with the object of applying the results of our investigations to the elucidation of insanity. It has long been matter of observation that the delirious and the insane appear to be in a dream; with this difference, that they *act* their dreams; whereas, in ordinary dreaming, the motor system never participates in the changes which the sensorial system undergoes. It is obvious that a knowledge of the state of the cerebrum in dreaming will very much facilitate a better understanding of the state of the cerebrum in insanity, and help to elucidate the much disputed question as to the pathology of that disease.

Two works have more particularly attracted our attention lately in reference to this subject, the titles of which we have given; they are both interesting publications. They have a further interest beyond this special subject, from the fact that two or three of Dr. Fosgate's countrymen have been journeying through this country for the purpose of lecturing on a pseudo-philosophy termed "electro-biology," and inducing a temporary condition in the cerebra of certain of their audiences closely analogous to, if not identical with, that more permanent condition which constitutes insanity on the one hand, and somnambulism on the other. It is not a little characteristic of the gullibility of the people at large, that numbers of individuals have been found, in our principal towns, willing to have their brain and mental powers subjected to the control of these itinerant strangers before numerous audiences, without any inquiry as to the probable results of these empirical proceedings on the delicate organ subjected to experiment. We have reason to think that the evil results of these practices are little known or even suspected. To tamper with the functions of so delicate and important an organ as the brain, simply for the gratification of a foolish curiosity, or the purposes of gain, is hardly less than criminal, and cannot be too strongly reprobated; nevertheless, the folly having been committed, we think it right to make the bane serve as the antidote, by also drawing some illustrations of the condition of the brain in sleep, dreaming, and insanity, from the *acknowledged* phenomena of this pretended science, as well as from the general literature of the subject.

In the first place, let us inquire what portion of the brain is involved in dreaming and in insanity; then, what is the mode of action of that portion, what are the phenomena manifested when its normal mode of action is disturbed, what are the agents of the change, and what practical conclusions may be deduced. We believe that there is no difference of opinion amongst physiologists as to what portions of the brain are affected in dreaming. Dr. Symonds, in his able and philosophical lectures, very concisely states the present views of the leading British neurologists. The abolition of sensation in profound sleep, and its modifications in dreaming, point to the nerves of sensation as being involved primarily in the change. These are connected, (as Dr. Symonds observes, in accordance with the views of Dr. Carpenter and others,) either directly, or through the spinal cord, with certain portions of the brain termed the sensory ganglia, the chief of which are the corpora quadrigemina and thalami optici. To these centres of sensation are conveyed the impressions made by outward agents, and here they probably become objects of consciousness. These sensory ganglia, therefore, have their functions modified in all those states of the cerebrum in which sensations, as *such*, are no longer felt—or, in other words, when the changes induced ordinarily in the sensory ganglia by impressions, are either not produced, although the impressions reach them, (and even pass through them,) or, if produced, do not become objects of consciousness. It is necessary to bear this principle well in mind, if we would perfectly comprehend the phenomena to be subsequently noticed—namely, that the impression is one thing, and the change induced in the sensory ganglia another—the two being as distinct as cause and effect; that if the faculty of *sensation* be abolished or modified, it is from some functional change in the sensory ganglia; that, nevertheless, the impressions may reach the ganglia, and the changes may be induced by them, yet the latter may not become objects of consciousness, *as sensation.*

Now, there are numerous animals which appear to have nothing superadded to these sensory ganglia; these constituting, in fact, the whole of their cerebrum. They live, therefore, simply a sensational life, and have probably little more knowledge of cause and effect than those animals who have only the sympathetic system of nerves. They have more extensive relations to external things—to the undulations, for example, of ethereal matter, which, reaching the auditory or optic nerve, cause auditory and visual sensations; and they *may* have a quicker perception of pleasure and pain; but their conservative and other instinctive acts are as mechanical and automatic as in the lowest tribes of animals. Their cerebral senses are superadded to strengthen and widen their *instinctive* life; they have no *manifest* mind, whatever may be the nature of that which regulates their actions so wisely and adaptively; and they have no true organ of mind. If a man is deprived of the organ of mind, or, what is the same thing, if its functions be abolished, those of the sensory ganglia continuing in activity, he would be, for the time, a *sensational* animal only, and his actions would correspond.

Now these sensory ganglia lying along at the base of the brain, are, in fact, only the portals to the portions of the brain resting upon them, and with which they are in intimate connexion. The mechanical apparatus—the mere *organ* of sense—collects and communicates the *right* impressions to the nerve, (for every nerve of sense has its own class of impressions, which alone it can receive, and none other,) and the nerve transmits them to the ganglion in virtue of certain changes induced in it by the impressions. Arrived at the ganglion, they begin from a new starting-point, as it were, and the ganglion is to the cerebral matter beyond what the nerve is to the ganglion. This is exactly the arrangement which exists inferiorly in the spinal cord, with reference to the nerves of touch and common sensation. Entering the grey matter of the cord, they carry impressions to it which excite changes therein—*sensational* in their character if the result of those changes is transmitted *upwards* as from a new starting-point — and automatically *motorial*, if, instead of so passing upwards, they are transmitted *across* the spinal cord into the motor tracts.

Where then, in this arrangement, are we to locate sensation? The more recent views of Dr. Carpenter are adopted by Dr. Wood, and have the merit of simplicity. Dr. Wood observes that there exist several centres,—namely, first, of muscular action in the spinal cord; secondly, of volition seated in the corpora striata and adjacent parts, having ample communications with the spinal cord downwards, and the cerebral hemisphere upwards; thirdly, a centre of sensation, independent like the others, but at the same time closely connected with the centre of volition; this is seated in the thalami optici and corpora olivaria, with which all the nerves of sense are more or less connected. These conjointly, Dr. Wood remarks, appear to form a ganglion for the sensations communicated by the nerves of touch, and therefore destined for the reception of sensitive impressions. The close association between them and the proper optic ganglia is explained by the close association between the senses of sight and touch, which is apparent both from the manner in which our ideas of external objects are communicated to us, and also from the joint operation of those senses in directing muscular movements. Fourthly, a centre of emotions, which is to be found in the mesocephale, its influence extending upwards to the hemispheres, backwards to the cerebellum, downwards to all the nerves of sensation and motion. Fifthly, another independent centre, seated in the cerebral convolutions, and the instrument of mental operations—namely, perception, memory, judgment, imagination, &c. In addition to these *five* centres (Dr. Wood only counts them as *four*) there is a centre for the combination of muscular movements, and another for respiration and deglutition. According to these views, (which are substantially those of Dr. Symonds,) sensation is seated in the olivary bodies and optic thalami. But what are we to understand by the term *sensation*, as used by Dr. Wood? This he nowhere defines, and we are inclined to think he has not a very exact notion of the sense in which he uses it, further than that sensation consists in "our ideas of external things." Let us take an example. Dr. Symonds states, that he remembers once in his sleep witnessing a prolonged storm of thunder and lightning, which he was afterwards able to trace to the light of a candle brought suddenly into the dark room where he had fallen asleep, and to the noise made in opening a door, the lock of which was never turned without a good deal of grating and rattling.

Now, it is obvious that in instances of this kind there is no sensation in the true meaning of the term, whether as restricted (with Dr. Carpenter, Dr. Bennett, and others) to the *consciousness of an impression*, or as including "ideas of external things." The impression of light and sound reached the sensorium, it is true, but not to excite sensations, inasmuch as a combined result followed, and series of associated ideas were excited, constituting the one idea of a thunder-storm. It was this idea of which the mind became conscious. Psychological phenomena exactly analogous are often observed in the delusions of the insane, as when pain in the epigastrium or uterus excites the delusion of a wolf, or fire, or serpent, being contained in the stomach or bowels, and the like. In all these cases the sensation is contained in the idea; and since the hemispherical ganglia are the centres of ideas of this class, we conclude that they also are amongst the seats of sensation.

Dr. Symonds thus points out the connexion of the nervous centres with sleep. When it is healthy and perfect, action is suspended in the sensory ganglia, the corpora striata, the cerebellum, a considerable portion of the hemispherical ganglia, or cerebral hemispheres, (some portion being employed in dreaming,) and those parts of the spinal cord which are used in the transmission of sensational impressions or volitional impulses. The medulla oblongata must not sleep, or respiration would stop. When the sleeper talks, the nerves which animate the vocal muscles are awake, and answer to the ideas and emotions developed in the hemispherical ganglia. In simple sleep-walking, or the minor degree of somnambulism, (the senses being still asleep,) the cerebellum is awake, and perhaps also the corpora striata in some degree, together with the related portions of the spinal cord. But in that form of somnambulism in which the subject of it sees and hears, though under the influence of the dream, the parts awake are the sensory ganglia, the corpora striata, portions of the cerebral lobes, the cerebellum, and the related portions of the spinal cord. This is an ingenious hypothetical organology, but a moment's consideration shows that it leaves a very large number of residual phenomena.

How is it, for example, (to mention one of many,) that the somnambulist sees and hears only in relation to his dream? Dr. Fosgate mentions an instance of this kind with which he was familiar. The subject, a merchant's clerk, was of a sanguineo-nervous temperament—irritable and timid. It was a favourite amusement with his fellow-clerks to commence a conversation with him (as soon as he was sufficiently asleep not to be easily aroused) relative to robbers breaking into the store-house. From his timorous disposition, this subject was undoubtedly on his mind when he retired to rest, and therefore could, by skilful management, readily be made the theme of his thoughts in sleep. By this management he could be induced to converse, leave his bed, dress, go into the street, and combat any person who should oppose him in the feigned character of a robber. On awaking he could relate nearly the whole transaction. Now, in this and similar examples the senses cannot be said to be shut, or sensation abolished. Touch, hearing, sight, are all open, but only to a *certain class* of impressions—namely, those which are in relation to the series of ideas constituting the dream.

It is evident, from all these considerations, that the writers before us have not attained to a true theory of sensation. Their views are, however, of a suggestive character, and in that respect useful. In the cases just mentioned an interesting analogy may be traced. It is well established that only certain kinds of impressions can be made on the sensory apparatus. Light and visual objects have no influence on the sense of hearing; simple or combined sounds cannot reach the sensorium through the nerves of vision. In like manner, when the right impressions have reached their respective ganglia, something more is wanting to the completion of the mental act which they excite, and they must go on or forwards to another portion or other portions of the nervous centres adapted for their reception, and ready to be influenced by them. No one sense is *solely* in operation during our waking state, except at the *moment* of the act of attention; the normal ideas which pass through the mind are seldom, if ever, compounded solely of the changes excited through one nerve of sense; unconsciously to ourselves we bring two or more into operation, and it is the combined or conjoint result in the brain which is presented to the mind, or of which, in other words, we become conscious. Hence if it so happen that this conjoint operation is prevented, by any change whatever in the cerebrum sufficient for the effect (as occurs in sleep, somnambulism, and insanity), the ideas are incongruous and imperfect; consequently the external world is not placed before the mind in its conjoint relations, and the perceptions are erroneous.

Where, then, we again ask, is the seat of sensation? To answer this question, let us more strictly define what is meant by the term. We have just used the term *perception*. Now perception is continually confounded with sensation by physiological and popular writers, and, without doubt, they are so closely allied, or so intimately connected with each other, that sensation and perception constitute one mental act. Still, philosophically, they must be discriminated. Sensation is applied to *feelings* irrespective of the cause. Perception includes not only the feelings, but the external object or thing causing the feelings. When the higher faculties of the mind are in operation on abstract ideas, the perceptions of them are conceptions, notions, &c. If an animal *feel*, it has been long supposed that it will manifest its faculty of sensation by corresponding movements; it was therefore an established proposition, that as certain adapted movements always followed the application of a stimulus to the nerves, those movements necessarily proved that the animal felt when the stimulus was applied. Hence an abundant source of error; for it has long been known to physiologists that certain movements of an admirably adapted character invariably result from simply acting upon the *material organ* of mind, or upon a portion of it, by *physical* irritants. For want of a better phrase, or from a reluctance to coin a new phraseology, this property of the organism was termed *corporeal* sensation—a contradiction in terms; nevertheless the doctrine was adopted by a metaphysical school, and the term sensation was used to indicate this *corporeal* sensation, or, in other words, to indicate the adapted and, *apparently,* rational respondence of the organism to certain impressions made on the nervous system independently of consciousness. It made no difference whether the impressions were made *directly* on the nervous centres, or, in other words, were *centric;* or whether they were *peripheral*—that is to say, reached the nervous centres from the surface of the body along the continuous nerve-fibrils running thence inwards.

Now, it has been fully shown, that these reflex movements are altogether independent of mind; that, in fact, the will and consciousness have nothing whatever to do with them. They not only go on when both the consciousness and will are abolished, but even after the head is separated from the body, and all mental action (if it be granted that the mind is seated in the brain) is rendered impossible. It has also been fully shown, and, indeed, is a matter within the sphere of any one's observation, that although the sensation or the feeling of pleasure or pain may accompany a movement thus excited, and occur coincidentally, the movement is not *caused* by the sensation, for we find that the sensation often induces us to exercise an act of intellectual will—or, in other words, to *control* the resulting movements. What really occurs when simple impressions are made on the nerves is this; if they be such as the impressed nerve is adapted to receive and transmit, they are received and transmitted to the ganglion or mass of grey matter in connexion with that nerve: by virtue of an innate property of the ganglion, certain movements result, which are such as are adapted to fulfil one or other of the instincts of the animal—namely, the preservation and well-being of the individual, the propagation of the species, and the protection of the young creature. The impression may be indicative of what will aid in the fulfilment of these instincts; in this case, if felt, it will excite *the sensation of pleasure,* or, if not felt, will excite movements which are observed to *accompany* the sensation of pleasure. On the other hand, should the impression be indicative of what will obstruct the fulfilment of those instincts, it will, if felt, excite the sensation of pain; or, if not felt, the *movements* which are known to accompany that sensation. In both cases, the movements or vital acts thus excited are conservative either of the organism or of the species—aiming to obtain that which is beneficial, or avert that which is injurious; but the whole takes place in the simplest forms, without any reasoning, or perception, or conception—there is no intellectual act—simply the feeling of pleasure or pain.

There can be no doubt whatever that in this series of vital changes there is a change or changes in the central ganglion, differing according as pleasure or pain is excited, and reacting on the motor apparatus in accordance with the changes—which (as we have seen) correspond to the necessities of the organism. The individual organism is not conscious of any change—it does not even know that there is a ganglion—it may not even be endowed with *self*-consciousness—yet the act of consciousness takes place; it becomes sensible of pleasure or of pain; and its vital mechanism is duly and properly put into motion. If we analyse the stages of this process we find that for its integrity it is requisite that the impression complete a circle—namely, from the surface to the sensory portion of the ganglion, thence through the ganglion to the motor portion, and from the motor portion to the vital apparatus or mechanism of the periphery. Now, the *act of feeling* takes place at the moment of centric action, or midway in the stage, when the adapted action is excited by the proper changes in the motor portion of the ganglion, and the impression passes over to it from the sensory portion, occupying but one moment of time. The proofs of these views are to be found in pathology and experimental physiology; we need only, as to the latter, refer to Grainger, Volkmann, Stilling, Van Deen, and others; as to the former, the works of numerous recent writers on diseases of the nervous system. In sleep, this circle is interrupted.

What, however, most merits the attention of the psychologist, is the great and fundamental principle of life, that all vital acts are adapted, that is to say, display the operation of that mental force which in man is termed reason, in animals, instinct. It is a remarkable circumstance that this principle and its laws of action have been almost altogether neglected by neurologists, by psychologists, and by the greater number of the students of metaphysics. This was not the method of the ancient philosophers, nor even of the fathers of modern metaphysics. To them, as well as the ancients, it was known as the hylozoic principle, or as *phusis*, (hence the primary meaning of the term *physiology* differs from the common meaning;) by the true Atheists, as the governing essence of the universe; by certain schools of Deists, as God, &c. We are satisfied the greatest elucidation of what are now impenetrable mysteries in mental philosophy may be derived from this quarter, so soon as physiologists have attained to the height of the great argument; and we may here observe, that henceforth it will be found impossible to limit the investigation of neurological phenomena to a study of the structure and functions of the nervous system—for this reason, that the phenomena we have just analysed take place in the same order or sequence in animals totally devoid of a nervous system, in germs of every kind, in vegetable life, and, in short, in every form of organism. The same great law is evidently in operation throughout animated nature; we see it in the laws of life; in the operations of instinct; in the works of reason. There is hardly an art or science which dignifies humanity, or ministers to the comfort and well-being of mankind, which is not to be traced in the operations of the blindly working "vital principle," or of the wonderful manifestations of instinct. Unconscious of the hidden bearings of his argument, Pope touches admirably on this connexion of reason with instinct.

> "Learn from the birds what food the thickets yield;
> Learn from the beasts the physic of the field;
> Thy arts of building from the bee receive;
> Learn of the mole to plough, the worm to weave;
> Learn of the little nautilus to sail,
> Spread the thin oar, and catch the driving gale;
> Here subterranean works and cities see;
> There towns aerial on the waving tree.
> Learn each small people's genius, policies,
> The ants' republic, and the realm of bees."

Universal science is pre-existent in nature; every branch of physics is practised by vegetable and animal organisms; not without a regard to the beautiful: hence our conviction that the whole range of psychological phenomena must be studied before we can lighten and enlighten

> "——— the burden and the mystery
> Of all this unintelligible world."

Now, the process, the analysis of which we have given, contains in it not only the basis of sensation and perception, but also of volition. In reasoning animals, the impression passes upwards to the hemispherical ganglia, and the act of *will* takes place at the moment the consequent movement is determined. At the same moment the act of perception also occurs, and the idea or consciousness of causation and the adaptation of the acts take place. At the same moment there is also *sensation;* but the sensation thus excited in combination with a perception, differs from what we may term a *primary* or *fundamental* sensation. To have what is termed the sensation of hardness or softness, it is requisite that the tactile apparatus be brought firmly in contact with a hard body, as marble—of roughness, or smoothness, that they should be brought firmly in contact by means of a similar, yet different adaptation of the muscular apparatus; and so the automatic, but conscious *use of the muscular system* must take place; otherwise there could be no perception of hardness or roughness, or the contrary. But the perception having taken place, the sensation which accompanies it may be resolved into a feeling of that which is congruous or incongruous with the well-being of the organism—the rough and hard being likely to be injurious, the smooth and soft the contrary.

As all perceptions may be pleasing or the contrary, there must be some mechanism in the cerebral hemispheres, as well as in the simpler central ganglia, whereby they are felt to be pleasing and displeasing; and this is another reason why we think sensation cannot be limited to the basilar ganglia of the encephalon. It is sufficient that there be already a condition of the cerebral hemispheres, such, that when new ideas are excited, the changes which accompany their excitation are found to be congruous or incongruous with the substrata already existent. This condition has been theorized on by various writers, but its true nature is not yet understood. All we can do to illustrate it is by the way of analogy. Just as we find creatures born into the world with their nervous system so constituted that certain impressions are painful, or the contrary, so from various causes the nervous system may become so constituted that certain ideas become painful, or the contrary. Amongst these causes are acquired habits, prejudices, established modes of thought—in short, all modes of mental and vital action, by which new substrata are developed. Ideas, if congruous with such, give pleasure, if incongruous, are painful.

Having thus cleared the ground, we are in a better position to understand what a sensation is and is not. Obviously, it is not merely the consciousness of an impression neither in theory nor in fact; equally obviously, it is the consciousness of a change, or of changes, produced in the organ of consciousness, directly or indirectly, by impressions, one or more. The point of consciousness is, in fact, that point in the general organ in which the changes take place, and may differ according to the nature of our sensations and perceptions. Hence it is possible to have a double and treble consciousness; to have also at one time pleasing, at another displeasing, sensations and conceptions from the same source. We are also in a better position to understand what consciousness is, and what relations it bears to the encephalic ganglia generally. We have said that it is necessary to sensation and motion that a circle in the nervous system be completed. Now, the body is a unit—it is indivisible—literally, an *individual.* It is true that limbs may be removed, and even the nervous system partially destroyed; still it is without injury to the unity of the consciousness, and simply from the fact, that the completion of the circle is in the sensorium. There every part of the body is represented, as it were, and although the mere mechanical apparatus may be removed, the vital unit remains intact, until the destroyer, entering the sanctuary of thought, crushes the admirable mechanism of the divine mind contained within, and the circle is rendered incomplete.

And so when deep sleep falls upon man, and he has dreams and visions of the night, the machine is thrown out of gear, and the circle is imperfect. The substrata of past thoughts are awakened into activity by various causes—sometimes ideas hid deeply in the caverns of memory are again developed, and vivid phantasms pass before the consciousness in infinite variety, the dreamer wondering whence he has got them, and puzzled with the fantastic tricks his memory and imagination play him. Impressions reaching the central axis from every point of the periphery, excite a thousand ripples (if we may be permitted the analogy) in the cerebral sensorium, each of which passes before the consciousness with inconceivable rapidity—undulating on ever, sometimes in well-ordered series of waves, so that connected thoughts arise in the mind with the precision of instinct, or in marvellous and incongruous combinations, with the effect to the mind's eye of a psychal kaleidoscope. That the cerebral matter is endowed with properties, such, that it is capable of these changes, is manifest enough from a consideration of the fact, that not only does the nervous organisation of perfect insects and vertebrates manifest phenomena equally wonderful, but even the amorphous microscopic germ contains within it, and dependent entirely upon the integrity of its organisation, that property whereby in well-ordered and admirably adapted sequence, the whole subsequent acts of the organism is performed.

Now what is the nature of the changes which occur in the cerebrum during dreaming, and its allied state, insanity? To determine this, we have to refer to the nature of the changes which occur normally in thought; and if we would ascertain this again, we must turn our attention to the changes which occur in the central axis of animals endowed only with instinct, and in the spinal cord during reflex acts. Now it is only by *analogy* we can in any way carry out these inquiries. Professor Gregory, indeed, hopes that, very shortly, clairvoyants will determine these identical questions by simple visual observation; that is to say, will be able to *see* the changes which occur in the brain in acts of thought; to us, however, this short and ready road is utterly closed; and as the microscope cannot aid us, and vivisections are useless, we have no other method than the method of *analogy*. Let us, then, pursue it in as simple a manner as we can. When the foot of a headless frog is irritated it is retracted, and the animal will even leap; if it be put into water, (provided the necessary conditions are attended to,) it will move its limbs as in swimming, and, in reality, the decapitated animal will swim. Now it is certain that, to perform these acts, a certain combination of numerous muscles is requisite, whereby their contractions are adapted to move the levers, (the bones to which they are attached,) so that the acts of retraction of the leg, and of swimming and leaping, will take place. It is equally certain that the volition or consciousness of the animal is not the power by which this necessary combination is effected; while it is also equally certain, from multitudes of experiments, that the power (whatever it may be) is in the nervous system, and particularly in the central ganglia, inasmuch as the *integrity* of that system is necessary to the production of the movement at all. In a conscious being, (as man,) an act of volition is the same, in one important point; for when a person withdraws his limb from an irritating agent, or leaps, or swims, he has no knowledge whatever of the muscles which he combines, or, indeed, of the fact that he has muscles at all. Muscular action has been likened to a performance on the pianoforte, and the mind has been represented as playing on the roots of the motor nerves, or their prolongation into the *corpora striata*, just as a performer plays upon the keys of a piano. How erroneous and useless the analogy is apparent enough; the mind simply *wills* the act, and it is done, provided the mechanism be in order; if that be deranged, it is not done. In like manner, when we reason, or, in other words, deduce the cause from the effect, the mind acts with the *rapidity* of instinct, and we even draw our conclusions, provided we have a perfect knowledge of the data, with the *precision* of instinct. This is almost paradoxical, but it is the fact, and so much so, that the mind thus acts even still more instinctively in its method, for it will pass through a whole train of thought, examine the premises, and draw conclusions, and yet the individual be quite unconscious of this operation of his own mind; so great is the analogy between the workings of instinct and of reason.

Although our views hardly bring us nearer to a knowledge of the nature of reason, will, and consciousness, they widen, to an infinite extent, the analogies by which we can determine their relations to the material organisation on the one hand, and the spiritual organisation on the other. The changes taking place in the central axis in the ordinary and multitudinous operations of instinct, have a distinct correlation with the changes which take place in the same axis in the multitudinous operations of thought.

If the mind begins to contemplate the results of an inquiry into the psychological nature of man from this point of view, and, ranging freely through natural history, collates the phenomena of mind as displayed in animated nature with the phenomena of the human will and consciousness, it is quickly lost in the infinite grandeur of the thoughts which the contemplation excites, as it wanders amidst that

> "Vast chain of being! which from God began,
> Natures ethereal, human, angel, man,
> Beast, bird, fish, insect, what no eye can see,
> No glass can reach.——"

The common source of the two great classes of rational and instinctive phenomena will strike the mind, and it will be led to the conviction, that by the most rigid induction man is shown to be made in the image of God, and is in very deed and truth a reflex of the Divine mind; that as immortality is the portion of his spiritual nature, and he will endure *to all* eternity, so the essence which constitutes his spiritual nature has been formed and developed *from* all eternity; that the faculties of instinct, acting blindly and without the self-consciousness of the individual, constitute the foundation and origin of the faculties of the reasoning mind; and that as *they* spring from the direct operation of the Divine mind, so also the conscious mind evolved from instinct, and rendered more and more perfect in intelligence and freedom of volition, springs from that direct operation also, and becomes finally conscious of its origin, and claims God as its Father. Having attained to this knowledge, with the same certainty of conviction as to the knowledge of its own existence, and of the fundamental axioms of physical and mathematical science, it has reached its *highest* knowledge.

All this may seem very foreign to our theme, but it is not so; for it is obvious, that as the phenomena of dreaming and insanity are of corporeal origin, or, in other words, result from changes in the functions of the cerebrum, it was necessary to hint what were the relations of the material organ to animated nature, on the one hand, and to mind, on the other. When persons speak of matter in relation to mental phenomena, they forget that the material organ is not made up of sticks and stones, and brute stuff, but is a most exquisite piece of mechanism, which no created thing can equal, and which it has been the work of ages to perfect. Without a due appreciation of this wonderful mechanism, all proper comprehension of mental aberration is impossible; unless the premises we have laid down be granted, all practical and useful inquiry into its laws of action is impossible.

Sleep depends on an altered condition in the functions of the cerebrum, whereby the impressions which reach it from all parts of the organism cease to excite those changes which constitute the *material portion* of the act of consciousness. Nevertheless, changes do occur in sleep; and these are of different kinds, according as sleep is more or less perfect. If consciousness be entirely abolished, the phenomena of the organism are those of vegetative life, and the man is simply a living machine, like animals devoid of feeling or consciousness. If, however, the sleep be imperfect, there is a very different condition, accordingly as the mind is conscious of the various changes going on, or according to the mode of action of the hemispherical ganglia. Perfect sleep, or the reduction of the animal to a vegetative mode of existence, is a great part of the scheme of Divine Providence, whereby animated nature is maintained in health and happiness. Dr. Symonds observes that little is known of the phenomena of sleep in the avertebrata; periods of inactivity with them are, perhaps, periods of sleep. But do not vegetables sleep? If we use the term in the wider sense of periodic inactivity of vital function, we cannot but agree with Linnæus on this point. In plants with compound leaves, at the approach of night the leaflets fold together, while the petiole is recurved, and the leaflets again expand and raise themselves at the return of day. The Hedysarum gyrans has ternate leaves, the terminal leaflet, which is larger than those at the side, does not move, except to sleep; but the lateral ones, especially in warm weather, are in continual motion, both day and night, even when the terminal leaflet is asleep. Plants are not only like animals in the general fact, but also in the details. Thus, as some animals sleep in heat and some in cold, some in the day and some in the night, so it is also with vegetables. The flowers of the crocus and similar plants expand beneath the bright beams of the sun, but close when they are withdrawn. So, on the contrary, the Œnotheras unfold their blossoms to the dews of evening, and wither away at the approach of day. The Victoria Regia sleeps during the day and wakes up at night, taking, like tropical animals, its siesta. During the late almost total eclipse of the sun, one of the specimens, now grown in England, awoke up and opened out its flower, as if it were night, imitating the reputed doings of animals under similar circumstances. These vital movements in plants are, perhaps, not all of the nature of sleep. Thus, the crocuses may shut up to protect the organs of reproduction from the cold and dews of night; but the day-sleep of the Victoria Regia lily, and of numerous other plants, appears to be a true sleep, having for its object repose from vital action. The sleep of tropical animals during the day is probably of a similar nature, as is also the prolonged torpidity of serpents during the heat of the tropical summer, and which seems to be a true hybernation, if the paradoxical phrase may be permitted. In the dormant condition of animals which are torpid during the winter, we have, perhaps, an exact illustration of perfect sleep. Not only are the animal functions brought into a state of complete inactivity, but even those of organic life are reduced to the lowest ebb compatible with the continuance of vital action. The respiration can scarcely be detected, and the circulation is wonderfully slackened. The pulse of the hamster beats in the ordinary condition at the rate of 150 strokes per minute; but in the hybernatory sleep it is only 15. Marmots make 500 respirations in an hour; when torpid, the rate is only 15. How nearly all bio-chemical action ceases during this state is shown by the fact that the temperature of the animals very nearly fall to that of the atmosphere; in ordinary sleep the temperature of the body is less from the same cause.

Trance-sleep is a *morbid* form of sleep, and has been frequently and fatally mistaken, it is to be feared, for death. In trance-sleep there seems to be the same suspension of the animal and organic functions as takes place in hybernation, but the hemispherical ganglia continue in intestine activity; and if all external perception be abolished, and the mind be exclusively conscious of the intestine changes, the thoughts and imaginations appear to the sleeper as perfect realities, and phenomena are manifested to the mind's eye which perhaps approximate as closely to the phenomena of the spiritual world and the realities of *pure* thought as any terrestrial phenomena can. The condition of the encephalon is very remarkable in all the forms of trance, (for there are several,) and is very worthy of special investigation. When the torpid state partially extends to the *motor* system, the *cataleptic* condition is induced—that is to say, the muscles contract automatically upon any slight impression being made on the surface, as on the muscles themselves by flexure of the limb, so that a l'mb or the whole body will remain for a prolonged period in the same position, or, in fact, until an impression from without alters the condition of the motor portion of the central axis. This is cataleptic trance. A more frequent form is that in which the muscular system is either entirely paralyzed, and the body is motionless, or else the motor system is in full activity, and the limbs and organs of motion generally respond to the ideas passing through the hemispherical ganglia: this latter form is somnambulism, the former is true trance. The so-called *magnetic* trance is simply somnambulism excited artificially by acting on the sensorium by a prolonged act of attention. There are several varieties of this form of sleep, from the profoundest insensibility to impressions, to a temporary and slight condition little differing from the waking state. The great difference between true trance and its modifications is this: that while the body is deprived of motor power, or, in other words, while the motor portion of the nervous system is not acted upon by the sensorial, the sensorial itself is fully active; and there are not only coherent dreams passing through it, as visions, &c., but the individual is conscious of his dreams on awaking, and can relate them. Trance, too, implies that the dreams have a special reference to the unseen world, in a religious sense.

In ordinary sleep there is also a difference of this kind observed. Persons on awaking may remember their dreams, or they may talk loudly, and appear much disturbed in their sleep, and yet have no recollection of the cause of their bodily movements, if awoke in the midst of the dream. In the latter instance, we must look upon the muscular acts as reflex cerebral phenomena. It is essential, perhaps, to the completion of our idea of a dream, that it be remembered; a somnambulist may enact a dream, and yet have no recollection whatever of anything he has done, although he may have been at work for an hour or two, or even more. In this case, he cannot correctly be said to dream; if, however, he recollects all that he has done, as if it had been a dream, then we may properly describe his condition as that of dreaming. The difference is not generic; nevertheless, it is of importance to remember these slight modifications of consciousness, in the form of acts of memory.

Dr. Symonds observes, that the simplest form of memory is the mere reproduction of a sensation, or the return of a thought, or of a former creation. When these occur by an effort of the will, the act of mind is termed *recollection;* when the past returns unbidden, or spontaneously, it is a *remembrance.* Both these states of the mind are dependent upon what is termed the *association of ideas.* As no change in the hemispherical ganglia can occur without an antecedent change as the agent, it is obvious that the association of ideas, considered physiologically, is dependent upon some *impression* made on the material organ of mind, of such a nature that it is adapted to re-excite the previously existing *material* ideas—a term that has often been used to characterise the *material basis* of the *true,* or metaphysical ideas. Now, of these re-exciting impressions there are at least two kinds, corresponding to the two forms of memory; the one dependent on the *will,* and arising immediately by its operation on some portion of the hemispherical ganglia; the other coming from without, and passing through the sensorial ganglia—the portals of the mind. In these two kinds of mnemonical impressions we have the analogues of the two kinds of material impressions—namely, that kind which excites movement immediately either with or without an accompanying sensation, and that which is exerted immediately by an act of volition, and is accompanied by a perception, or conception. In other words, the impressions in acts of memory are excito-sensorial, or volitional-sensorial; but (just as in reflex acts) they require a pre-existent substratum, or otherwise the act of memory never takes place. The re-excitement of sensations, in remembrance, is a curious illustration of this analogy; for not only will the ideas be reproduced in connexion with it, but also the muscular acts and other changes which the original sensation excited. Instances of this kind are very familiar, as when vomiting is excited by the recollection or remembrance of a disgusting object, drug, &c. Mere acts will often be re-excited in this way, with hardly any consciousness of the impression, if they have been so often performed as to become habitual—and sometimes habitual movements will be re-excited by almost any impression. We have occasionally experimented on a costermonger, who sells sugar-plums at the corner of a street we pass daily, and who continually repeats, "ha'penny an ounce—two a-penny." If, when he is quiescent, we excite his attention by a stare, or a frown, or a peculiar smile, he will immediately utter his cry. Analogous to this is the case of the merchant's clerk we have quoted from Dr. Fosgate's work, whose fellow-clerks, acting upon his known habits of thought, excited a dream at will, and made him enact it.

The suggestion of dreams may therefore be dependent upon internal or external impressions, or upon both combined. The internal impressions may either be *ideas*, or trains of ideas, excited by the intestine changes in the hemispherical ganglia, or *sensations* excited by impressions derived from the viscera and sensorial nerves. The external impressions may be derived from various objects that excite the organs of sense, not causing, however, either sensations or perceptions, but re-exciting conceptions only, or trains of ideas. The larger proportion of our revived conceptions have reference to visual objects, and these re-excite other conceptions in sequence, or association. When the friend of bygone times, Dr. Symonds observes, revisits us in sleep, we do not recognise his form merely as one that had been seen before, but with its presence return some, at least, of the occurrences in his life, the points in his character, his sentiments, and his familiar talk. For, so far is it from being true, that visual images alone are produced in dreams, that it often happens that the remains of several sensations are simultaneously renewed.

There is a great variety in the mode of reproduction of ideas in sleep. They may arise in their exactly pre-existent form, or they may have a kaleidoscope arrangement; and fragments of many may be patched together in a mosaic, which to the dreamer appears perfectly natural and possible, but to the waking reason is nothing but the grossest absurdity. It is a remarkable circumstance, that this state occurs in insanity as well as in dreaming. The wildest incoherences, the confounding of personal identities, the mingling of material and mental properties, the most miraculous violations of the best ascertained laws of nature, excite no more surprise or wonder than the commonest events of life. In the following example, mentioned by Dr. Symonds, the practitioner experienced in insanity will recognise delusions which occasionally occur to the insane. "A gentleman to whom this institution is largely indebted, gave me the following experience :—'I have several times appeared to read a portion of an imaginary work, as *regularly* as if it had been real. I have also dreamed that I was dead, and that I carried my own body in a coach to bury it; and that when I reached the place of burial, a stranger said, 'I would not advise you, sir, to bury your body in this place, for they are about to build so near it, that I have no doubt the body will be disturbed by the builders.' 'That,' I replied, 'is very true; I thank you for the information, and I will remove it to another spot;' upon which I awoke.'" We were informed by a friend that he had a dream of a somewhat similar kind. Returning, much fatigued, from a ball, where he had taken an unusual amount of liquids, and, finally, some diuretic champagne, he quickly fell asleep, and dreamt that he was in a large ball-room, and that one of the company created a considerable disturbance by his wandering about the reception-rooms for a convenient place to relieve a pressing want, and attracting the notice of the company to his unusual conduct. He behaved so improperly, that the dreamer found it necessary to remonstrate with him very strongly on the impropriety, not to say immodesty, of his behaviour, and was in the act of doing this, when he awoke to find that it was himself upon whom nature called so imperatively for relief. When conversations are held with persons in dreams, there is the same duplicate consciousness; and in insanity and delirum no phenomenon is more common. We have a curious case under our notice at present, of an epileptic youth of eighteen, whose paroxysms occur at intervals of four weeks, and are accompanied for two or three days by a particular form of delirium approaching insanity. He wanders hither and thither after the fit, apparently without an object; and on being asked the reason, says there is a man in his head, who says he must do so and so, and he is obliged to do it. If he reads while under the epileptic influence, he says the man in his head repeats everything he reads, "loud up;" and often when he is not reading, the man will "talk a deal of nonsense about Kentucky." In this case there is, doubtless, a double consciousness, one portion of the cerebrum being in a dream.

A very curious thing is, to dream that one is dreaming; but we believe it is not so very uncommon. Dr. Fosgate relates a dream of this kind which occurred to himself on the night which followed the committal to paper of certain views of his touching mysterious and prophetic dreams :—

"Not long since," he observes, "I was examining the Croton

Waterworks, in New York city, including some pits which were open in the streets where the great iron trunks were exposed, and on the occasion alluded to, my mind was in part occupied with this subject. On falling asleep, I dreamed that in passing one of the pits, I jumped down upon a tube about three inches in diameter, for the purpose of inspecting the work more minutely; but when in this position, on casting my eyes below, an awful chasm presented itself, crossed in various directions by huge iron water-tubes, but the bottom was invisible. However, the depth was ninety feet. In what way the information was imparted was indistinct, but such appeared the awful depth under my slippery footing. I could just barely reach the surface above, but could lay hold of nothing, and therefore attempted to leap to the top. I failed, and in falling, lodged upon the place I just left. This fall will never be forgotten, so long as excessive fright, commingled with horror, can leave an impression on my mind. I then thought to cry for help, but dared not, lest my feet should slip, and precipitate me down the dark chasm beneath. After reflecting long upon my perilous situation, I commenced feeling around the platform surrounding the top, and finally succeeded in fastening my fingers in a crevice between the planks, by which means I drew myself up. The dream, ordinarily, would have ended here; but my mind now turned upon the subject which had occupied my attention the preceding evening until a late hour. I thought, in my dream, that what had just transpired was a prophetic dream, and to what it might point my reflections were directed, as well as to what would be the best course to elude the impending danger. During these reflections I awoke, excessively exhausted. In this instance, in a dream, I dreamed that I was dreaming."

It will be seen that there were two distinct dreams in this instance, and the analysis of them is not without interest. The exciting cause of the dream was, probably, that state of the cerebrum which is induced by looking from a height in the waking state, but which will occur in the insane, and especially as a monomania, or as a passing symptom, in persons who have severely exercised the organ of thought. This had probably been the case with Dr. Fosgate; during sleep, the intestine changes in the cerebrum had produced the idea in an organ already predisposed from excessive action, and the material idea, so excited, led to the train of ideas constituting the dream. But the sensation of danger of falling from a height was only momentary in duration, and ended with the supposed fall; when it ceased, the mind became occupied with the means of escape, and, finally, the whole passed away into a new association of ideas, namely, that which had already occupied the mind in reference to the prophetic character of dreams.

Dr. Fosgate's dream was at the commencement simply *incubus*, a form of dreaming respecting which we are surprised to observe that writer remarks, that "no satisfactory explanation of the phenomena has been given, all being mere speculation, not founded on facts." We always considered that many of the various forms of incubus originated in disorder or disease of the thoracic and abdominal viscera, but principally of the heart and lungs. The instinct of love of life, or self-conservation, is most usually excited by a painful sensation, originating in a morbid condition of the blood dependent on disease of these viscera, and indicating, in fact, a state in which life is really imperilled. The whole series of phenomena usually resolves itself into an *imperfect depuration or aëration of the blood.* Now this may take place from various causes. There may be disease of the kidneys or liver, causing retention of urea or of bile in the blood, or there may be disease of the heart; an overloaded stomach may press upon it, or upon the lungs; or the bedclothes or part of the dress may be pressing upon the mouth or throat, so as to interrupt respiration; or there may be functional disturbance of the heart's innervation by disease or predisposition to disease, of the medulla oblongata; or the fibres of the heart may have undergone the fatty degeneration, so that the paralysis of the voluntary motor system, which characterises true sleep, and which implicates also the cardiac movements so much as to render them slower, absolutely takes effect upon the weakened fibres, so as to seriously impede the circulation. Without doubt, fatal fits of apoplexy, induced in this way, have been preceded by incubus; and its frequent recurrence must be considered to be a very serious symptom.

Incubus attacks children of a nervous temperament and irritable fibre, and is well known to nurses as "the megrims." In these it seems to be connected with gastro-intestinal irritation, remotely and primarily—and, secondarily and proximately, with *slight* spasm of the glottis, or a *momentary* and imperfect attack of laryngismus stridulus. It is probable that *any* painful sensation, or even morbid impression, originating in the viscera, and influencing, by incident excitor action, the circulation, so as to interrupt the due aëration of the blood, or any sufficiently toxic condition of the blood of a depressing kind, will induce one or other form of incubus or frightful dreams. It is noticeable, too, that whatever may be the cause of the *sensation* of horror, the dream has almost always reference to danger to life. In the case of Dr. Symonds' friend, who "awoke one morning desperately clutching and tugging at the strings of his night-cap, having been dreaming that a viper had fastened upon his throat, and he was doing his best to tear it away," it is probable that the strings had not only irritated the skin of the throat, but compressed the larynx, so as partially to interrupt respiration. The dreamed cause of danger, and the accompanying scenes of horror, will depend entirely, as to their character and specialities, upon the idiosyncrasies of the individual—his habits of thought, of study, of life. A lady under our care, with cancerous disease of the uterus and appendages, who suffers much from pain in the hypogastric region, describes her dreams as being of the most horrible description. Being religiously disposed, and having been long a member of a strictly religious society, her dreams of horror most frequently turn upon religious phantasms; spectral images, clothed in white yet with black faces, groaning horribly, sulphureous smells, fearful thunderings and flashings, and even hell itself, in all its horrible realities, disturbing her nightly rest with terrors indescribable.

"The dreamer," Dr. Fosgate observes, "often believes himself shipwrecked, and left to the fury of the winds and waves; or he is fast approaching the brink of a dreaded precipice, without the power to turn aside, and over which he must unavoidably fall; or he is pursued by wild beasts intent on devouring him, and through all he feels spellbound, and unable to help or defend himself; he struggles with all his power to be released from this frightful situation, but apparently to no purpose, until at last, when he considers his destruction inevitable, a sudden bound frees him from his condition, and a dream is disclosed, which he believes to have been the cause of his sufferings."

A very interesting fact is, that the same cause will produce a similar incubus-phantasm in the dreams of several persons, thus setting aside the marvellous points in the coincidences of so-called prophetic dreams. Thus we read lately of a whole regiment starting up in alarm, declaring that they were dreaming that a black dog had jumped upon their breasts and disappeared, which curious circumstance was explained by the discovery, that they had all been exposed to the influence of a deleterious gas generated in the monastery in which they were sleeping.

We might quote from Dr. Fosgate's description of incubus, as a disease (for such it is), with satisfaction to our readers, for the writer of it has suffered from its attacks since his earliest remembrance, and most graphically depicts its course. In his case it appears to be connected with *centric* disease, and hence, probably, his exclusive pathology. "This disease," he remarks, "we consider to be purely nervous. The attendant dyspnœa and congestion are its consequence, and not the cause, as has been believed and supported by pathologists"—a proposition much too general, as the fact just mentioned shows.

It is interesting to observe, that the imperfect respiration and obstructed aëration of the blood which accompanies advanced phthisis and intense or extensive bronchitis, rarely induces incubus. Still, in those states of the lungs the respiratory mechanism has a peculiar and characteristic action, for the patient moans physically or automatically, and sometimes so loudly as to excite a sympathising dream in his own mind, or even to awake himself. The sound is peculiarly distressing, being the tremulous moan of intense grief or sorrow, and often causes a harrowing conviction in the mind of the affectionate watcher, that the mental and physical sufferings of the patient are great. Yet they are not; for the sound depends on reflex action, and is only the mechanical groan of suffering nature; no sensations of pain are excited, and consequently the hemispherical ganglia are not thrown into dreams and terrors of the night. Incubus (in this wide sense of a painful modification of the conservative instinct) is allied, on the one hand, to two peculiar forms of epilepsy, on the other, to the varied forms of melancholia. Certain epileptic patients raise a cry of terror just previously to the convulsions, which is certainly automatic and reflex, since they are not aware themselves that they raise the cry, nor feel any particular dread. Again: epileptics will occasionally start off in the greatest terror, and run as if escaping from a fearful pursuer, (exhibiting the form termed *epilepsia dromica,*) until arrested by the convulsions; yet they also are usually without knowledge of the sensation which induces the flight, except, in some instances, they observe that they are seized with an indescribable dread. Hippocrates seems to have been aware of this relation between incubus and epilepsy: in his "Treatise on Epilepsy" ("the Sacred Disease") he observes, "I have known many persons in sleep groaning and crying out, some in a state of suffocation, some jumping up and fleeing out of doors, and deprived of their reason until they awaken, and afterwards becoming well and rational as before, although they be pale and weak," &c.*

There is a form of painful dream which may be confounded with incubus, and, without doubt, is allied to it—namely, that which depends upon pain in some part of the body, (an illustration of uterine pain, as a cause, has been given,) although it is especially pain in the skin to which we allude. In this, also, the conservative instinct is roused into action, yet with a difference. Dr. Reid relates of himself that the dressing of a blister which he had applied to his head becoming ruffled so as to produce pain, he dreamt that he had fallen into the hands of a party of North American Indians, who were scalping him. Dr. Beattie states that once, after riding thirty miles in a very high wind, he passed a night of dreams, which were so terrible that he found it expedient to keep himself awake, that he might no longer be tormented with them.

The relation of incubus to melancholia is less direct and will have our notice further on. We will rather consider the relations of painful instinctive or emotional sensations to our dreams. It has often been

* The Genuine Works of Hippocrates, p. 843. Sydenham Society's edition.

noticed, that under certain conditions, not always well understood, the complexion of the dreams is diametrically opposite to the waking thoughts in persons suffering in body or mind. The prisoner for life enjoys freedom, happiness, and home in his dreams; the famished man feeds to fulness; the thirsty man drinks to satiety. Mr. Moffat thus describes his dreams after toiling through the deserts of Africa:—

"We continued our slow and silent march. The tongue cleaving to the roof for thirst, made conversation extremely difficult. At last we reached the long wished-for waterfall; but it was too late to ascend the hill. We laid our heads on our saddles. The last sound we heard was the distant roar of the lion; but we were too much exhausted to feel anything like fear. Sleep came to our relief, and it seemed made up of scenes the most lovely. I felt as if engaged in roving among ambrosial bowers, hearing sounds of music, as if from angels' harps. I seemed to pass from stream to stream, in which I bathed, and slaked my thirst at many a crystal fount flowing from mountains enriched with living green. These pleasures continued till morning, when we awoke speechless with thirst, our eyes inflamed, and our whole frames burning like a coal."

Intense grief and other emotions will excite the opposite states; even "Joy has its tears, and transport has its death." Sir W. Scott felt this on the death of Lady Scott. Describing his state, he remarks—"Gay thoughts strangely mingled with those of dismal melancholy; tears which seemed ready to flow unbidden, smiles which approached to those of insanity."* The same antagonistic condition takes place in persons given to strong devotional exercises, of whatever sect they may be. Hence the temptations of Romish ascetics and *ecstatica;* hence the "buffetings of Satan" of many of Wesley's converts; hence their paroxysms of involuntary laughter during their religious exercises, their maniacal oaths and blasphemies during the fit. This polarity, if the phrase may be permitted, which is thus operative in dreams, is seen, in a simpler form, in the waking dreams of the so-called clairvoyants of the mesmerists, or the "sensitives" of the Baron Von Reichenbach. We find such persons repeatedly describing objects in an inverted or wrong position, mistaking the right side for the left, speaking of a river flowing north to south, instead of south to north, describing the points of the compass erroneously, &c. In certain dreams this perversion of the ideas is seen in the wrong notion the sleeper has of his position, imagining he is upside down, and seeking to rectify the error by placing his head at the foot of the bed and his feet on the pillow. This is by no means an unusual circumstance in nurseries. Occasionally an analogous condition occurs in delirium. We lately met with a case

* Memoirs, by Lockhart, vol. vii. p. 10

of this kind in the fourteenth volume of the "London Medical and Physical Journal," in the person of a female subject to paroxysms of hysterical delirium, who, during the attacks, could not resist the impulse to place the chairs upside down, which she did because in their ordinary position they appeared to be inverted. She also laughed heartily, and expressed her surprise at seeing the attendants, as she thought, standing on their heads. The polarity of insane notions is the most interesting, however, in connexion with this subject. It has long been a matter of common observation how completely the moral character of the individual is changed, or certain of his ideas monomaniacally perverted. Thus, the man who "rolls in wealth" goes about wringing his hands, under the impression that he is a pauper, and will die in a workhouse; or, *vice versâ,* the pauper calmly dispenses untold gold and estates of infinite extent to his attendants, or such of his brother patients as have won his esteem. In like manner the gentlewoman of highly cultivated manners, irreproachable modesty, perfect truthfulness, and the sweetest temper, under certain forms of functional disease of the cerebrum, will become absolutely the reverse; that is to say, coarse in manners, immodest, singularly deceitful, cruel, malicious. We cannot but hope that the closer study of the phenomena of dreaming may throw some light on these interesting cases; and we indulge a hope that some intelligent student of psychology will work out the instructive analogies we have mentioned to a full elucidation of the question. Dr. Symonds takes a passing notice of the analogy, in this respect, between insanity and dreaming—of "that curious suspension of the moral sense which is sometimes experienced" in dreaming. "Deeds from which we should shrink with horror when awake, are performed, not only without the least remorse, but even without any question in our mind as to their propriety."

The state of the *intellect* in dreaming is the next point to consider. The late Dr. Binns well and graphically describes the state of the mind in dreaming. It becomes inventive, and discovers new places, new forms of things, and novel modes of sensibility. It conceives, fancies, or creates, associates and combines objects; sometimes incongruous and discordant, sometimes natural and normal; often exquisite and beautiful; but more frequently horrible and repulsive. We see huge monsters, vast plains, innumerable armies, indescribable creatures, transcendent beings, unimagined forms, inscrutable chasms, stupendous mountains; or we witness astounding prodigies. We perceive the sun and moon on our right hand, the stars on our left, the elements, fire, air, earth, and water, at our feet, and the glory, and the brightness, and the brilliancy of ten thousand thousand meteors above our heads. We hear, we talk, we move; walk, run, swim, fly. No obstacles arrest, no impediments obstruct, our progress; space, time, and probability are annihilated.*

Well may Dr. Binns remark,

"We believe that dreaming and insanity are nearly allied; for maniacs are inundated with a flow of thoughts, a superabundance of ideas, and a catenation of impressions, which invert order, escape arrangement, and defy control, exactly similar to images in dreams. Their cerebral organs riot in confusion; they exhibit brilliant and burning flashes of wit, but they are lost in the coruscations which follow; they enjoy glimpses of elevated genius, but the prospect is soon obscured; they sometimes reason acutely, but their premises are confounded; they talk eloquently and write vigorously; but their images are unconnected by detail, their reasoning unsupported by evidence, and their arguments unrestrained by any rule of precedent, mode of thought, or law of logic. Is not this the case in dreams?"†

It will be practically useful to notice in detail some points in the intellectual state during dreaming, with reference to the more permanent and morbid, but analogous condition in insanity. We have already noticed the curious suspension of the moral sense during the dreaming state, but, in reality, the whole of the instinctive and emotional faculties are perverted; and if we were to go through the various forms of *moral insanity* and melancholia, we should find a perfect parallelism. "The pacific," Dr. Symonds remarks, "become pugnacious, the gentle and open-hearted entertain strange suspicions and animosities; and the pure give utterance to sentiments which should be like the snatches of old songs that fall from the innocent lips of Ophelia." Doubtless, this abolition of the moral sense depends upon the same cause as the total want of perception, that the *acta* of the dreams are utterly incongruous. The ideas themselves seem to originate in the same way that various fleeting ideas of the same kind occur in the waking state, and are known popularly as "temptations of the devil," and are premonitory of impulsive insanity. To have dreams of a vicious and wicked character, is no proof that the individual is vicious or wicked, secretly or openly, as some have supposed.

A general sense of vivacity and pleasure, or a feeling of depression, is felt in health by many persons. Hardly any one, indeed, is exempt from an alternation of these states. They are reproduced or felt in dreams; and often an individual will awake cheerful and happy, or oppressed with an indescribable sense of oppression, without being able to remember any particulars of the dreams, or the dreams themselves, except that a dream has been dreamt.

* The Anatomy of Sleep. By Edward Binns, M.D. 2nd edition, p. 39.
† Ibid., p. 180.

"Dreams in their development have breath,
And tears, and tortures, and the touch of joy;
They leave a weight upon our waking thoughts,
They take a weight from off our waking toils,
They do divide our being. * * * * * *
* * * * They have power,
The tyranny of pleasure and of pain."

That these feelings are less dependent on the dreams than by a bodily condition on which the waking feelings of sadness, or the contrary, depends, is proved by the facts—that very often what would be distressing events to us if really occurring, excite no emotion when *dreamt* to occur; and that the state of mind felt during dreaming continues after waking, and can be traced to functional or structural disease of one or other of the viscera.

A very remarkable circumstance, and an important point of analogy, is to be found in the *extreme rapidity* with which the mental operations are performed, or rather with which the material changes on which the ideas depend are excited in the hemispherical ganglia. It would appear as if a whole series of acts, that would really occupy a long lapse of time, pass ideally through the mind in one instant. We have in dreams no true perception of the lapse of time—a strange property of mind! for if such be also its property when entered into the eternal disembodied state, time will appear to us eternity. The relations of space as well as of time are also annihilated, so that while almost an eternity is compressed into a moment, infinite space is traversed more swiftly than by real thought. There are numerous illustrations of this principle on record. A gentleman dreamt that he had enlisted as a soldier, joined his regiment, deserted, was apprehended, carried back, tried, condemned to be shot, and at last led out for execution. After all the usual preparations a gun was fired; he awoke with the report, and found that a noise in an adjoining room had, at the same moment, produced the dream and awakened him. A friend of Dr. Abercrombie's dreamt that he crossed the Atlantic, and spent a fortnight in America. In embarking, on his return, he fell into the sea, and awaking in the fright, found that he had not been asleep ten minutes.

"The rapidity of mental action occurring in dreams," Dr. Fosgate observes, "where events, which in their actual development would occupy hours, days, nay, even years, are compressed and comprehended in a few minutes, or even seconds, is finely illustrated in the dream of Count Lavalette:"—

"'One night,' he says, 'while I was asleep, the clock of the Palais de Justice struck twelve, and awoke me. I heard the gate open to relieve the sentry, but I fell asleep again immediately. In this sleep I dreamed that I was standing in the Rue St. Honoré, at the corner of the Rue de l'Echelle. A melancholy darkness spread around; all was still. Nevertheless, a low and uncertain sound soon arose. All of a sudden I perceived, at the bottom of the street, and advancing towards me, a troop of cavalry; the men and horses, however, all flayed. The men held torches in their hands, the flames of which illuminated faces without skin, and with bloody muscles. Their hollow eyes rolled in their large sockets, their mouths opened from ear to ear, and helmets of hanging flesh covered their hideous heads. The horses dragged along their own skins in the kennels, which overflowed with blood on both sides. Pale and dishevelled women appeared and disappeared at the windows in dismal silence; low inarticulate groans filled the air, and I remained in the street alone, petrified with horror, and deprived of strength sufficient to seek my safety in flight. This horrible troop continued passing in rapid gallop, and casting frightful looks at me. Their march, I thought, continued for five hours, and they were followed by an immense number of artillery waggons, full of bleeding corpses, whose limbs still quivered. A disgusting smell of blood and bitumen almost choked me. At length the iron gate of the prison shutting with great force awoke me again. I made my repeater strike: it was no more than midnight; so that the horrible phantasmagoria had lasted no more than *ten minutes*—that is to say, the time necessary for relieving the sentry and shutting the gate. The cold was severe and the watchword short. The next day the turnkey confirmed my calculations. I, nevertheless, do not remember one single event in my life, the duration of which I have been able more exactly to calculate.' "

This remarkable relation of the lapse of time to the intestine changes of the hemispherical ganglia in thought, is a tempting subject for speculation. We forbear, however, preferring to note an analogous relation which occurs in certain morbid conditions of the brain, of equal interest psychologically. It has been noticed in cases of impeded aëration of the blood from strangling or drowning, and occupies that short moment of vital action between the commencing transmission of carbonized blood to the brain and the abolition of consciousness. It may also be of *emotional* origin, and there are *toxic* cases of a similar kind on record. Dr. Fosgaté has very judiciously directed attention to this analogy, as it regards the *emotional* cause, and illustrated it by cases. He says—

This "rapidity of mental action is often experienced on occasions of great personal danger, and almost always turns upon a review of the *past life* of the individual, in which incidents the most trifling are brought distinctly before the mind, which occurred at remote periods, and each circumstance in the order of its occurrence. This has often been experienced in falls from elevated positions, as the roofs of buildings, which could have occupied but a very few seconds of time in the descent. An old sea-captain once related to me that during a fall from the rigging of a vessel, from which he barely escaped destruction, he distinctly remembered every act of his life, even the purloining of fruit from the neighbouring orchards, and the depredations upon hen-roosts, as well as the maternal admonitions inflicted for his juvenile delinquencies."

Dr. Binns relates a very interesting example of this kind of rapid mental action, and (of course) molecular change in the material organ; it has, indeed, a double interest, inasmuch as it also illustrates the analogy, on the one hand, between ordinary dreaming and that condition of the brain alleged to be mesmeric, which is the proximate cause of "clairvoyance," as it is termed, and the so-called higher phenomena, and, on the other, between these states and ecstatic trance, monomaniacal visions, and mania.

"We are acquainted," Dr. Binns states, "with a gentleman, who being able to swim but little, ventured too far out, and became exhausted. His alarm was great; and after making several strenuous but ill-directed efforts to regain the shore, he shouted for assistance, and then sank, as he supposed, to rise no more. The noise of the waters in his ears was at first horrible, and the idea of death, and such a death! terrific in the extreme. He felt himself sinking, as if for an age, and descent, it seemed, would have no end. But this frightful state passed away. His senses became steeped in light. Innumerable and beautiful visions presented themselves to his imagination. Luminous aërial shapes accompanied him through embowering groves of graceful trees, while soft music, as if breathed from their leaves, moved his spirit to voluptuous repose. Marble colonnades, light-pierced vistas, soft grassy walks, picturesque groups of angelic beings, gorgeously plumaged birds, golden fish that swam in purple water, and glistening fruit that hung from latticed arbours, were seen, admired, and passed. Then the vision changed, and he saw, as if in a wide field, the acts of his own being, from the first dawn of memory to the moment when he entered the water, grouped and ranged in the order of the succession of their happening, and he read the whole volume of existence at a glance; nay, its incidents and entities were photographed on his mind, limned in light, and the panorama of the battle of life lay before him. From this condition of beatitude—at least, these were the last sensations he could remember—he awoke to consciousness, and consequently to pain, agony, and disappointment."

In Everett's life of Dr. Adam Clarke, the Wesleyan commentator, and a great linguist, there is an auto-biographical account of his sensations when drowning; and it is remarkable that there was the same feeling of tranquillity and pleasure as described above:—

"At first, I thought I saw the bottom clearly, and then felt neither apprehension nor pain; on the contrary, I felt as if I had been in the most delightful situation; my mind was tranquil and uncommonly happy. I felt as if in Paradise. * * I cannot recollect that anything appeared defined, nor did my eye take in any object, only I had a general impression of a green colour, as of fields or gardens. But my happiness did not appear to arise from these, but appeared to consist merely in the tranquil, indescribably tranquil, state of mind."

Dr. Adam Clarke does not describe that rapid perception of past events, or, in other words, that comprehensive *act of memory*, whereby the actions and doings of the individual in days long gone from the recollection, are vividly recalled to remembrance; the condition of his nervous system was, in fact, analogous to that induced by hachisch, opium, nitrous oxide, &c., of which this vivid memory is but an accessary part. The English opium-eater mentions an instance in which this most remarkable psychological phenomenon was fully developed. "I was once told," he observes, "by a near relative of mine, that, having in her childhood fallen into a river, and being on the very verge of death, but for the critical assistance which reached her, she saw *in a moment* her whole life in its minutest incidents arranged before her simultaneously, as in a mirror, and she had a faculty developed as suddenly for comprehending the whole and every part."

A literary friend, objecting to our views, directs our attention to Clarence's dream in King Richard III., as described by Shakspeare. The immortal dramatist is almost always true to nature, and is so most particularly in this particular instance. It is a *dream* of drowning, and not the reality; hence the phenomena are described as those of *incubus*, because the conservative instinct is aroused. Still, there is the dreaming similarity between the reality and the illusion kept up with admirable tact and truth to nature. Clarence is in prison, and dreams of escape:—

"Methought that I had broken from the Tower,
And was embarked to cross to Burgundy;
And in my company my brother Gloster,
Who from my cabin tempted me to walk
Upon the hatches; thence we look'd toward England,
And cited up a thousand heavy times,
During the wars of York and Lancaster,
That had befall'n us. As we paced along
Upon the giddy footing of the hatches,
Methought that Gloster stumbled; and in falling
Struck me, that thought to stay him, overboard,
Into the tumbling billows of the main.
O Lord! methought what pain it was to drown!
What dreadful noise of water in mine ears!
What sights of ugly death within mine eyes!
Methought I saw a thousand fearful wrecks,
A thousand men that fishes gnawed upon;
Wedges of gold, great anchors, heaps of pearl,
Inestimable stones, unvalued jewels,
All scattered in the bottom of the sea."

Then, when Brackenbury asks him if he were not awakened "with this sore agony," Clarence replies (and herein Shakspeare shows his matchless art and powers of observation) in terms which indicate that there was the act of memory, like that described above, but dream-like, and not tinged with pleasure, but with pain, such as must necessarily accompany incubus in all its forms:—

> "O no! my dream was lengthened after life;
> O, then began the tempest of my soul!
> I passed, methought, the melancholy flood,
> With that grim ferryman which poets write of,
> Unto the kingdom of perpetual night.
> The first that there did greet my stranger soul,
> Was my great father-in-law, renowned Warwick,
> Who cried aloud, '*What scourge for perjury*
> *Can this dark monarchy afford false Clarence?*'
> And so he vanished. Then came wandering by
> A shadow like an angel, with bright hair,
> Dabbled in blood; and he shrieked out aloud—
> '*Clarence is come—false, fleeting, perjured Clarence—*
> *That stabbed me in the field by Tewksbury;*
> *Seize on him, furies! take him to your torments!*'
> With that, methought a legion of foul fiends
> Environed me, and howled in my ears
> Such hideous cries, that with the very noise
> I, trembling, waked; and, for a season after,
> Could not believe but that I was in hell;
> Such terrible impression made my dream."

This whole description is true to nature, even to the last line. The impressions of a vivid dream often dwell in the mind for some time after waking, and leave the individual in doubt whether they are phantoms or realities.

This recal of past events to the memory, in dreams and in morbid conditions of the brain, is a singularly suggestive fact. It indicates the power of *mind*, in the abstract, to comprehend, with a faculty little short of omniscience, the meaning and significance of those minute mysterious changes in the material organ which constitute the physical basis of dreams. It indicates, also, the immense capabilities of matter, in being rendered subservient to such remarkable spiritual phenomena. But when we pass from the creature to the Creator; when we contemplate the endowments of the Supreme Mind,—of "the Father of the spirits of all flesh,"—as manifested in His offspring, we feel that we can almost understand how, just as the physical changes in the material organ, passing through their phases, in one moment reveal the doings of years, so, also, the doings of all created things, past and present, may be revealed to the glance of the Infinite, in virtue of the minute physical changes His will directs; and so we get a glimpse of the *possibility of omniscience.*

On the other hand, the mind is struck with wonder at the singular powers with which creative mind has endowed matter. The microscopic—the infinitely minute—changes which it passes through in acts of thought, and especially in the acts of memory we have described, are more utterly beyond our comprehension, and, indeed, more grand, because more inexplicable, than the vast changes in the relations of the masses which roll through infinite space in "cycle on epicycle." They reveal to us phenomena belonging to matter when it is conjoined with and the instrument of mind, which alter and decompose all our ordinary ideas of its properties, to the development of entirely new conceptions. Lamartine must have had reflections of this kind when he wrote the following, on watching the insect life and motes and particles of matter rendered visible in a sunbeam—"D'insectes colorés, d'atomes bleus, et d'ailes." It is amongst the grandest touches of philosophical poesy.

"Comme ils gravitent en cadence!
Nouant et dénouant leurs vols harmonieux!
Des mondes de Platon on croirait voir la danse,
S'accomplissant aux sons des musiques des cieux.
L'œil ébloui se perd dans leur foule innombrable;
Il en faudrait un monde à faire un grain de sable;
Le regard infini pourrait seul les compter.
Chaque parcelle encore s'y poudroit en parcelle.
Ah! c'est ici le pied de l'éclatante échelle,
Que de l'atome à Dieu l'infini voit monter.

Pourtant chaque atome est un être!
Chaque globule d'air est un monde habité!
Chaque monde y régit d'autres mondes peutêtre,
Pour qui l'éclair qui passe est un éternité!
Dans leur lueur de temps, dans leur goutte d'espace,
Ils ont leurs jours, leurs nuits, leurs destins, et leur place,
La pensée et la vie y circulent à flot;
Et pendant que nôtre œil se perd dans ces extases,
Des milliers d'univers ont accompli leurs phases.
Entre la pensée et le mot!"*

Nor are these reflections, as to the nature of mind on the one hand, and of matter on the other, in what may be termed their physical or natural relations, less interesting than the *moral* considerations of the subject. Dr. Symonds notices these by a passing remark, observing—"It is a fearful liability of our nature to have the past summoned before us, when we have fondly hoped that it was hid for ever in deepest night—to anticipate what is to occur hereafter—

'Each faintest trace that memory holds
So darkly of departed years,
In one broad glance the soul beholds,
And all that was at once appears.'"

When the condition of the cerebrum, which is the proximate cause of these phenomena, occurs permanently and morbidly as *insanity*, it must be a fearful state of suffering, if the dark side—the painful instead of the pleasurable—be developed. Fortunately, the painful is rare, or temporary, and only when there is concurrent corporeal disease of some part of the body, giving the character of incubus to the re-excited or newly developed images; the pleasurable condition is the

* Jocelyn, tom. i. p. 196, 12mo. 1838.

more frequent, as if Providence mercifully tempered the wind to the shorn lamb.

Another remarkable circumstance in dreaming is, that often all our fundamental ideas become *infinite*, as it were, for hardly another word will characterize those which pass through the mind. This fact as to *time* has been already fully shown; it is exactly the same with *space, number, extension*, &c. In one of the dreams described by De Quincey, he felt that

"The sense of space, and, in the end, of time, were both powerfully affected. Buildings, landscapes, &c., were exhibited in proportions so vast as the bodily eye is not fitted to receive; space swelled, and was amplified to a sense of unattainable infinity. This, however, did not disturb me so much as the vast expansion of time. I sometimes seemed to have lived for seventy or one hundred years in one night; nay, sometimes had feelings representative of a millennium passed in that time; or, however, of a duration far beyond the limits of any human experience."

It has occurred to ourselves to experience this expansion of the fundamental ideas during dreaming. Being feverish one evening, we saw innumerable rows of *tinsmiths* or blacksmiths, hammering furiously in row upon row, each row prolonged apparently *ad infinitum* into space, and each individual hammering with might and main most indescribably swift. The cause of the dream was the fall of a fire-iron in another room. When we dozed again, the shadows thrown on the wall of the room gradually shaped themselves into gigantic forms, and even the figured stripes on the Marseilles quilt assumed the appearance of the most beautiful classic statues, so that the whole appeared like Parian statuary of exquisite proportions, only the lower extremities were indefinitely prolonged into a rounded mass. We know a gentleman who has occasionally analogous ideas in the waking state—a species of delirium—only he is quite conscious and rational at the time. Poisons of the narcotic kind have occasionally a similar influence. This is particularly the case with hachisch, or extract of hemp. To the individual who has taken it for the purposes of pleasurable intoxication, minutes seem hours, and hours are prolonged into years. M. Moreau (who has investigated the psychological relations of the drug*) mentions, as an illustration, that when under the influence of a moderate dose, it seemed to him as if two or three hours had passed when he had made but a few steps in the passage of the opera-house, and as he advanced, the passage seemed interminable, its extremity receding as he pressed forwards. Frequently, when walking along the Boulevards, persons and things at a certain distance presented the same aspect as if he had viewed them through the large end of

* Du Hachisch et de l'Alienation Mentale, Etudes Psychologiques. Paris: 1845.

an opera-glass, thereby suggesting the idea of increased distance. The *Amanita Muscaria*, an intoxicating fungus, causes the person under its influence to take a stride sufficiently long to clear the trunk of a tree, when he wishes to step over a small stick; alcoholic drinks have occasionally the same effect.

These phenomena may all be recognised in various forms of insanity. Ideas of untold wealth, of estates comprising tens of thousands of acres, and the like, are very common. So, also, ideas of space and time are modified, inducing the most singular delusions. Coleridge's "Kubla Khan," although a dream, is just what an insane person might have written, and manifests the expansion of the ideas of space and number very clearly. It also shows other curious points in psychology —as, for instance, rhythmical alliteration.

> "In Xanadu did Kubla Khan
> A stately pleasure-dome decree;
> Where Alph, the sacred river, ran,
> Through caverns measureless to man,
> Down to a sunless sea.
> So twice five miles of fertile ground
> With walls and towers were girdled round;
> And here were gardens bright with sinuous rills,
> Where blossom'd many an incense-bearing tree;
> And here were forests, ancient as the hills,
> Unfolding sunny spots of greenery.
> But, oh! that deep romantic chasm which slanted
> Down the green hill, athwart a cedarn cover!
> A savage place! as holy and enchanted
> As e'er beneath a waning moon was haunted
> By woman wailing for her demon-lover!
> And from this chasm, with ceaseless turmoil seething,
> As if this earth in fast thick pants were breathing,
> A mighty fountain momently was forced;
> Amid whose swift half-intermitted bursts,
> Huge fragments vaulted like rebounding hail,
> Or chaffy grain beneath the thrasher's flail;
> And 'mid these dancing rocks, at once and ever,
> It flung up momently the sacred river.
> Five miles meandering with a mazy motion,
> Through wood and dale the sacred river ran;
> Then reached the caverns measureless to man,
> And sunk in tumult to a lifeless ocean;
> And 'mid this tumult Kubla heard, from far,
> Ancestral voices prophesying war."

The dream-poem of Coleridge reminds us of two other striking and most interesting peculiarities of dreams—namely, first, that they are sometimes *prophetic*, and, secondly, that they are occupied with acts of *pure reason* and intellect. These two occasional peculiarities of dreams are, indeed, but variations of the same fundamental condition, as a few observations will show. The assistance supposed to be furnished in sleep towards the solution of problems which puzzled the waking reason, or to the sleeper in anticipating some coming event, or attaining to some knowledge unattainable when awake, has given rise to various superstitions and psychological theories. A modification of the same condition in hysterical women, and somnambulistic and cataleptic persons generally, constituted the psychological basis of pagan oracles, and the various forms of divination by crystals, &c.—a subject which merits special notice. Some philosophers, struck by the remarkable nature of the phenomena, (as Sir Thomas Browne and Addison,) have been induced to suppose that the soul in this state is partially disengaged from the body, and therefore more intelligent — a doctrine which (as Locke observes) "every drowsy nod shakes." Illustrations of the general fact abound. Franklin stated to Cabanis, on several occasions, that he had been assisted in his dreams in many affairs. Condillac, while writing his "Cours d'Etudes," was frequently obliged to leave a chapter incomplete, and retire to bed; but on more than one occasion he found, on awaking, that it was finished in his mind. Condorcet, upon leaving his deep and complicated calculations unfinished, after having retired to rest often found their results demonstrated to him in his dreams. Voltaire, like La Fontaine, composed verses frequently in his sleep, which he remembered on awaking. Johnson states that he once, in a dream, had a contest of wit with some other person, and that he was very much mortified by imagining that his opponent had the better of him. In the late Dr. Wigan's work on the "Duality of the Brain," there are some excellent illustrations of this morbid state, as it occurs in the insane. Dr. Fosgate justly observes, that the wonderful clearness of the mind in dreams must have been observed by all who have given attention to the subject. This lucidity is particularly observable in imaginary conversation, public speaking, and composing, the minutiæ of which the mind seldom retains on awaking. It is certainly probable that this mental clearness depends upon the passive condition of the external senses, which modifies the impressions of external things that would otherwise divert and divide the attention. We have, in the state of *abstraction*, or deep thought, a condition not far dissimilar from sleep, inasmuch as the mind thereby avoids all disturbing impressions, and so follows more closely the current series of ideas,—which, moreover, are developed in more direct and more connected sequence, than when waves of confusing ideas excited by various external impressions impinge upon them. It is for this reason that the student seeks undisturbed quiet, and rejoices in a freedom from the distraction of mind which externals excite. When the *ideagenous* changes in the hemispherical ganglia go freely on in regular association and sequence, all external impressions being shut out, except those which are congruous with the ideas in connexion with the changes, the tissue is in a state analogous to that in which *instinctive* operations take place. Now we have already referred to this state, and have observed, that even when we reason, the mind acts with the rapidity of instinct, and we often draw conclusions before we have any *conscious* knowledge of the premises; in short, that in the *waking* state we often think, and yet not become conscious of the course, or even the result, of our thoughts.

Any one may ascertain the truth of this statement for himself by carefully analysing his own thoughts. On investigation, he will also be astonished how little attention has been directed to a careful observation of the more minute phenomena of mental action. One illustration we may mention by way of example. Often a person will feel unhappy and depressed, he knows not exactly why; only he has an uneasy anticipation of something disastrous or unpleasant. If, in this state, he analyse his corporeal and mental condition, he will either find that bodily disease excites the "thick-coming fancies," or, what is more usual, some circumstance has happened which is likely to influence his future unfavourably,—being unconscious all the while that he had already weighed the probably disastrous or unpleasant results. Dr. Symonds quotes a very fitting illustration of these views, from the autobiography of Captain John Crichton.

From all these considerations it is obvious, that in a prophetic dream a person may have the conclusions of waking thoughts (he having deduced them unconsciously) re-excited and made manifest to his consciousness in a dream, under which circumstances they will appear new. Or the thoughts may actually occur during the dream, as if in the waking state, at the same time becoming objects of consciousness—yet instinctively and automatically, and therefore with the precision of instinctive reasoning.

It is in this way, we suspect, that dreams have proved prophetic. *Prescience,*—one of the most striking and inscrutable of the *instinctive* faculties,—is also that which is most commonly in operation in instinctive life. Hence it is not remarkable that that faculty which dominates amongst all the instincts of irrational creatures, should re-appear in the human organism when it is thrown by suspension of the cerebral senses into the irrational condition. It seems strange that organized matter should have this innate prescience, but it is manifest throughout nature, from the evolution of the germ and the anticipatory formation of the organs necessary to successive phases of existence, to the prudent foresight of adult life. We may well ask, with Pope,

> "Who taught the nations of the field and wood
> To shun their poison and to choose their food?

*Prescient*, the tides or tempests to withstand,
Build on the wave, or arch beneath the sand?
Who made the spider parallels design,
Sure as De Moivre, without rule or line?
Who bid the stork, Columbus-like, explore
Heavens not his own, and worlds unknown before?
Who calls the council, states the certain day?
Who forms the phalanx, and who points the way?"

If, then, this anticipation of the future be so universally manifest in organized matter that there is no exception, can we, with any inductive propriety, except the *organism of man* from the universal law? We apprehend not. The simple fact that all nature anticipates a real future, is, indeed, the strongest argument in natural theology for the reality of a future state; because, since that anticipation is innate in organisms as a law of *their* being, so it must needs be innate in man as a law of *his* being. And in what clime or region is man without a hope of a future life?

"Lo the poor Indian! whose untutored mind
Sees God in clouds, or hears him in the wind;
His soul proud science never taught to stray
Far as the solar track or milky way;
Yet simple *nature* to his hope has given,
Behind the cloud-topp'd hill, an humbler heaven."

The apparently prophetic anticipation of events in dreams is, then, a natural phenomenon, and so far from being closely allied with the *purely* spiritual world in causation, it depends upon the special exercise of one of the most common, if not the most universal, of *instincts*. Our knowledge of the inner workings of organisms in reference to *apparently* rational prescient acts, and of the relations of the cerebro-spinal or central axis to the instinct, in animals endowed with nerves and central ganglia, is so utterly imperfect, that we can advance no further, hypothetically, than the principles we have laid down. In a vast majority of prophetic dreams, the whole of the facts are not stated; consequently, it is not possible to trace the dream-ideas to their sources; and even if they were, it would still probably be impossible, because (as we have already shown) the mind may compare and deduce, and establish a conclusion, of which it does not become conscious until the whole series of ideas are acted in the dream. Consequently, results and events may be thus unconsciously anticipated in the waking state which reappear as things *done* in a dream. For this reason, dreams of this kind should not be always neglected.

Certain forms of delirium are analogous to prescient dreaming; and in certain states of the cerebrum the prescient instinct seems to be developed, as, for example, during the closing scene of life. Aretæus describes this state as supervening on the delirium of kausos or brain-fever. He states, that on the subsidence of the violent excitement, there arises a state in which the patient's mind becomes clear, and all his sensations exquisitely keen; that he is the first person to discover that he is about to die, and announces this to his attendants; that he seems to hold converse with the spirits of those who have departed before him, as if they stood in his presence, and that his soul acquires a prophetic power. With all the appearance of conviction as to the truth of these statements, Aretæus observes that although the bystanders fancy the patient to be rambling and talking nonsense, they are afterwards astonished at the fulfilment of the prediction. It was a notion entertained of old, and has been transmitted down to us from the earliest records of mankind, that a prophetic power attends man's last hour. We find instances in Holy Writ, as of the dying Jacob. In the sixteenth book of the "Iliad," Patroclus prophesies the death of Hector, and in the twenty-second, Hector prophesies the death of Achilles by the hand of Paris, at the Scæan gate. Shakspere also makes John of Gaunt prophesy, in Richard the Second, who exclaims—

> "Methinks I am a prophet new inspired,
> And thus, expiring, do foretell of him."

In this instance, and in others of poetic origin, the prophecies have no speciality of detail; they rather point out probabilities as deduced from past events. It is the wisdom of a sound judgment, exalted in its manifestations by the morbidly exalted condition of the organ of thought, which we see in action—it is simply a state—

> "When old experience doth attain
> To something like prophetic strain."

Allied to this is another class of phenomena. It is probable that those sudden perceptions of truth, and long sought-for-relations, which often come upon the mind like inspirations, are due to an action of the cerebrum in thought, analogous to that which occurs in dreams. We more particularly refer to instances of this kind like that which occurred to Archimedes, when he ran from the bath crying *eureka*, after the sudden solution of the problem which had occupied his thoughts. Such sudden perceptions of hidden relations, or of truths, most frequently occur immediately on awaking from sleep, before the senses are quite open to external impressions.

These modes of cerebral action and the concomitant spiritual acts—like the acts of memory which take place during sleep, or under certain cerebral states, and like the development of infinitely grand ideas under like circumstances—are singularly suggestive to the psychological inquirer. What, he will ask, if it be the lot of man to attain to a prescience in the moral and spiritual world, similar to that which is manifested by irrational beings in the animal and instinctive world? What if in a higher stage of development the *future* be presented to his mind's eye with the vividness and omnipresence with which he occasionally regards the *past?* Strange, indeed, it is, that the marvels of instinct-mind, have never been considered to be typical (as we believe they are) of the true and ordinary laws of the human mind, when, emancipated from the chrysalis state of this life, it has attained to its perfect stage of growth.

*Special faculties appear* to us to attain to an extraordinary degree of development in dreams; this, however, is no proof that such degree is attained. Dr. Symonds very justly observes, referring to the want of *taste* in our dreams, "the most miserable doggerel may then pass before the mind as exquisite poetry. Orations may seem to be uttered worthy of the lips of Demosthenes, and arguments may be maintained which seem as irrefragable as the demonstrations of Euclid; and yet, were these reasonings and declamations uttered by a waking person, they would sound little better than the incoherent ravings of a maniac. Yet, even to this general rule," Dr. Symonds adds, "there have been remarkable exceptions. Cases are on record of judges, who in their sleep have delivered decisions of the weightiest kind; and of poets, who in that state have composed verses of great power and beauty, though they were by no means exempt from a certain degree of mystical indistinctness. The most striking instance is Mr. Coleridge's poem, entitled Kubla Khan, which he himself characterised as a psychological curiosity." We have already quoted this curious poem, but we do not think it by any means a solitary example. Eminent composers have composed very sweet airs in their sleep. The best proofs, however, of this remarkable development of special faculties are presented in certain forms of somnambulism, in hysterical delirium, and in the so-called clairvoyant state. Dr. Fosgate mentions specially that form of somnambulism in which there is sleep-*talking,* rather than sleep-*walking,* and the subjects of which are familiarly denominated "sleeping preachers." In this class of cases it is only that we have one kind of dream acted, instead of another; the individual delivers a sermon or an oration, instead of writing a book, or enacting some busy scene of active ordinary life. The case reported in the great French Encyclopædia, and which was observed by an Archbishop of Bourdeaux, is of this kind. It is that of a young ecclesiastic who was in the habit of getting up during the night, in a state of somnambulism, of going to his room, taking writing materials, and composing and writing sermons. He also wrote pieces of music in this state. Mr. Spencer met with a young female whose paroxysms of somnambulism were modified by the prescient instinct, so that she prophesied in them. One of John Wesley's assistants used to preach in his sleep.

From phenomena not widely dissimilar from these, ancient nations were led to the induction that the *insane* were *inspired* by the Divinity, and revealed the future, or were possessed of a wisdom not their own. It is from this notion, traditionally handed down from the earliest ages, that in the East to this day the wandering maniac has a certain reverence and kindness shown to him. The psychiatric practitioner cannot fail to have met with numerous examples of this special development of the faculties, one or more, in the insane; indeed, the instances are so numerous and common, that we need not further refer to the fact.

What is the state of the brain in sleep, dreaming, and insanity? We have already referred to the anatomy and physiology of the cerebrum in relation to this question; we have now to examine the metaphysical side. In the first place, it is of primary importance to observe, that the consciousness of the individual is variously modified in the three conditions. This is a fundamental fact in the inquiry. In addition to the ordinary phenomena of sleep and dreams, we have those morbid analogous states, induced by so-called mesmeric, or electro-biological agencies, and the morbid modifications of the will, and consciousness induced therein. No one can peruse Dr. Bennett's and Dr. Wood's statements without noticing the great similarity between these states, on the one hand, and insanity, delirium, and dreaming, on the other. The various processes by which these conditions are induced are now well understood; they all essentially consist in inducing a *continued act of attention*, more or less prolonged, in proportion as the cerebrum of the individual is more or less predisposed to be acted upon, or, in other words, fatigued. It has long been known to physicians and metaphysicians, that during each act of attention the consciousness (for attention is an operation of the consciousness) is directed solely to the one object which is the subject of the act, so that, for the time, the individual is insensible to all other impressions whatever. Now total insensibility is profound or perfect sleep; hence consciousness is abolished during that state; if, then, we could, after concentrating the consciousness upon a single object, permanently fix the necessary changes in the cerebrum which render the perception existent or present to the mind, impossible, we abolish the consciousness. It follows, therefore, that the morbid abolition or modifications of consciousness referred to, are not dependent upon a morbid condition of the spiritual element, whatever that may be, but upon a morbid condition of the organ. Dr. Fosgate has touched upon this view of the etiology of sleep. After mentioning the various modes recommended for inducing sleep, he observes, that they all depend upon some plan whereby the mind is ultimately brought to a single idea, monotonous in character, and there steadily held until sleep is induced by a normal act of our constitution. An act of attention withdraws the mind from external impressions; but external impressions are withdrawn from the mind. Thus quietude and darkness are usually sought by the sleeper; and abstraction of mind from the current of thoughts. Rhythmically recurring impressions will also so modify the cerebrum, that the consciousness is changed. Undulating sounds and sights, a droning voice, gentle friction, "mesmeric" passes, and the like.

The best methods of inducing sleep, however, are those by which the individual withdraws the consciousness from external impressions; for, practically, the means are in his own power. "If I could arrest," says Catlow, "the attention of any of my audience, so that he would think of nothing but what I was doing at the moment, I could prick him with pins without his feeling it. And if the act of attention were continued too long—longer than is compatible with the individual constitution of the mind—I could suspend the sensibilities altogether, and produce sleep, which varies according to the impressions on the senses through which I isolate or monopolize the attention." This is precisely the method of causing mesmeric sleep, or the sleep of hypnotism. All that is wanted is a long-continued act of attention—either continually gazing at an object—*any* object, or listening to the same sound, or rather, to the same sounds continually repeated, or repeating, continually, the same words, according to Macnish's method, namely, repeating internally any well-known rhyme, or repeating the alphabet backward, or feeling the same frequently-repeated touch, as a stroking. If it be wished to produce somnambulism or trance, the method must be modified. In this case the attention must be withdrawn from external objects, just as when it is wished to induce sleep, but the mind must be kept active, in a given course of thought. Thus, if spectral illusions be wanted, the individual must first be prepossessed with the idea that he is about to see spectral appearances; and if they are wanted to be of a definite character, the mind can be directed in the principle of suggestion and association to the appearance desired; this being done, he should look intently at something adapted to the excitation of the visual sensorium, as brightness, or its opposite, blackness. For the former, crystals, glass, mirrors, water, the reflection of the sky in water; or, for the latter, any black thing, as a drop of ink, will be the best. To attain to the so-called higher phenomena, a training of the hemispherical ganglia is all that is necessary, provided the individual be already predisposed to irregular nervous action: this is attained simply by frequent repetition of the process, and when the proper morbid condition is excited, the infinitely various modifications of mental phenomena which belong to dreaming, delirium, somnambulism, trance, &c., may, one or other, be induced.

And INSANITY *may also be thus induced*—not temporary insanity alone, but the reality—a sad reflection! On this point we cannot do better than quote Dr. Bennett's observations:—

"The great object of all who seek proper self-education is, to control the emotions and passions, and regulate the imagination by the severer faculties of judgment—comparison and attention. Hitherto, medical men, so far from exciting, have done all in their power to prevent such phenomena; but now it has been clearly shown that they may be produced in numbers of people by the ignorant and mercenary, there is too much reason to fear that nervous disorders will increase among us. It is well known that cases are on record of individuals who, commencing by the imitation of hysterical or epileptic convulsions, have at length found themselves really labouring under these diseases; nor is it unreasonable to suppose, that the mental faculties will be greatly injured in persons who frequently surrender up their own wills, and act in accordance with the extravagant ideas suggested to them. After all, the pleasure of excitement principally consists in feeling that it can be regulated, and is under command. The moment it ceases to be so, a sense of the imperfection becomes most agonizing to the mind, and gives rise to that despondency so common among the insane."

Without doubt, the sleeplessness which precedes an outburst of insanity is a cause of morbid cerebral action, as well as an effect. Opiates have been found very beneficial in arresting the progress of the disease; but all practical men must acknowledge, that they give them with some fear lest the desired result may have substituted for it, an additional degree of morbid action. In a better knowledge of the physiology of sleep and dreaming, we may find a better means of throwing the brain into repose, than in the administration of narcotics. Now, the modifications which the cerebrum undergoes in acts of attention are allied to sleep, and recent investigations, conducted as well by enlightened physiologists as by ignorant empirics, tend to show that we may be able to acquire such a knowledge of the *physiology* of attention and of consciousness generally, as to apply the knowledge easily, pleasantly, and safely, to the treatment of cerebral disease, more particularly as manifested in the various forms of insanity and delirium.

